# Determining the Risk of Type 2 Diabetes for rs1801133 Genotypes in Multiethnic Populations: A Global Meta-Epidemiological Study

**DOI:** 10.3390/ijms26093987

**Published:** 2025-04-23

**Authors:** Fahrul Nurkolis, Nurlinah Amalia, Yosi Yohanes Putra Tandi, Ariq Fadhil Athallah, Muhammad Reva Aditya, Ammar Nojaid, Farizky Martriano Humardani, Achmad Fabiansyah Prapriatna, Nurpudji Astuti Taslim, Dante Saksono Harbuwono, Raymond Rubianto Tjandrawinata

**Affiliations:** 1 Faculty of Medicine, Universitas Airlangga, Surabaya 60132, Indonesia; fahrul.nurkolis.mail@gmail.com; 2 Medical Research Center of Indonesia, Surabaya 60281, Indonesia; 3 Institute for Research and Community Service, State Islamic University of Sunan Kalijaga (UIN Sunan Kalijaga), Yogyakarta 55281, Indonesia; 4 Medical Study Program, Faculty of Medicine, Universitas Brawijaya, Malang 65145, Indonesia; 5 Master Program of Biomedical Science, Faculty of Medicine, Universitas Brawijaya, Malang 65145, Indonesia; 6 Department of Internal Medicine, Dr. Cipto Mangunkusumo National Central Hospital, Jakarta 10430, Indonesia; 7 Doctoral Program in Medical Science, Faculty of Medicine, Universitas Brawijaya, Malang 65145, Indonesia; 8 Division of Clinical Nutrition, Department of Nutrition, Faculty of Medicine, Hasanuddin University, Makassar 90245, Indonesia; 9 Division of Endocrinology, Metabolism and Diabetes, Department of Internal Medicine, Faculty of Medicine Universitas Indonesia, Dr. Cipto Mangunkusumo National Referral Hospital, Jakarta 10430, Indonesia; 10 Metabolic, Cardiovascular and Aging Cluster, The Indonesian Medical Education and Research Institute, Faculty of Medicine Universitas Indonesia, Jakarta 10430, Indonesia; 11 Center for Pharmaceutical and Nutraceutical Research and Policy, Faculty of Biotechnology, Atma Jaya Catholic University of Indonesia, Jakarta 12930, Indonesia

**Keywords:** type 2 diabetes, rs1801133, CC genotype, methylenetetrahydrofolate reductase, multiethnic group, metabolic syndrome

## Abstract

The rs1801133 (C677T) polymorphism of the methylenetetrahydrofolate reductase (MTHFR) gene has been linked to type 2 diabetes (T2D) risk. This study aimed to assess the association between rs1801133 genotypes (CC, CT, TT) and T2D across multiethnic populations and to identify genotype- and region-specific risks. A global meta-epidemiological analysis was conducted using data from 19 studies comprising 6479 participants from Asia, Africa, Europe, and America. Odds ratios (OR) and 95% confidence intervals (CIs) were calculated using random-effects models. Subgroup analyses by region were also performed. The results of the CC vs. CT dominant genetic model were OR 95% CI = 0.63 (0.46–0.87); *p* = 0.005; the CC vs. TT genetic recessive model yielded OR 95% CI = 0.59 (0.38–0.91); *p* = 0.02; and the CT vs. TT codominance genetic model yielded OR 95% CI = 0.95 (0.65–1.37); *p* = 0.78. Based on the subgroup analysis, the CC genotype is predominantly associated with an increased risk of T2D in both Africa and Europe. From this study, the CC genotype was proven to be highly contributory to T2D risk compared to the CT and TT genotypes. These findings highlight the need for ethnicity-informed genetic screening and targeted prevention strategies in global diabetes management.

## 1. Introduction

Type 2 diabetes (T2D) is one of the most prevalent metabolic syndromes on a global scale. It is caused by a state of insulin resistance leading to high blood glucose levels. According to the World Health Organization (WHO), approximately 830 million people worldwide have diabetes, with the majority living in low- and middle-income countries. In Asia, T2D is most prevalent in China (88.5 million individuals) and India (65.9 million individuals) due to their large population sizes [1]. Together with its chronic progression, T2D often manifests based on a complex interaction between its modifiable (such as diet, tobacco use, and physical activity level) and non-modifiable (such as age, genetics, and ethnicity) risk factors [2]. 

Several studies have highlighted alterations in genetic substances—such as nucleotide sequences, which may lead to changes in protein—that directly influence signaling process and lead to the development of diabetes. The methylenetetrahydrofolate reductase (MTHFR) enzyme plays a role in the metabolism of homocysteine and folate by catalyzing the conversion of 5,10-methylenetetrahydrofolate to 5-methyltetrahydrofolate. It has been established that a genomic polymorphism, specifically the MTHFR C677T (rs1801133) transition mutation from C→T at exon 4; 677 nucleotide, plays a significant role in the development of T2D. This transition mutation leads to a substitution of the amino acid alanine with valine, resulting in an impaired and thermolabile form of the enzyme [3]. 

This genomic polymorphism has been found to have a stronger association with T2D in the Asian population than in the Caucasian and African populations [4]. Environment and genetics might contribute to such differences. Therefore, it is important to validate the relationship between this genetic polymorphism and T2D across different areas and ethnic groups in order to achieve a more effective therapeutic outcome [5]. This meta-analysis was conducted to determine the significance of three rs1801133 genotypes (CT, CC, TT) associated with the development of T2D in the global population. Moreover, considering the interactions between genetic predisposition and modifiable lifestyle factors such as diet, physical activity, and micronutrient intake, integrating a lifestyle medicine perspective may enhance risk prediction and support targeted prevention strategies for T2D [6,7,8].

The primary objective of this study was to conduct a global-scale meta-epidemiological analysis to investigate the associations between the rs1801133 polymorphism and the risk of T2D across diverse multiethnic populations. The secondary objectives were to identify genotype-specific risks, particularly the role of the CC genotype, previously considered protective, and to explore regional variations that may inform population-specific genetic screening and precision medicine approaches. The findings of this study aim to support the development of targeted, genomics-informed prevention strategies, particularly in low- and middle-income countries with a high diabetes burden and limited genetic infrastructure. Beyond its academic contributions, this study has substantial implications for global health by advocating for population-specific genetic screening and precision medicine approaches. It provides a critical foundation for policymakers and healthcare systems to develop targeted, genomics-informed prevention strategies—especially in low- and middle-income countries.

## 2. Result

### 2.1. Study Selection and Identification

**Figure 1:**
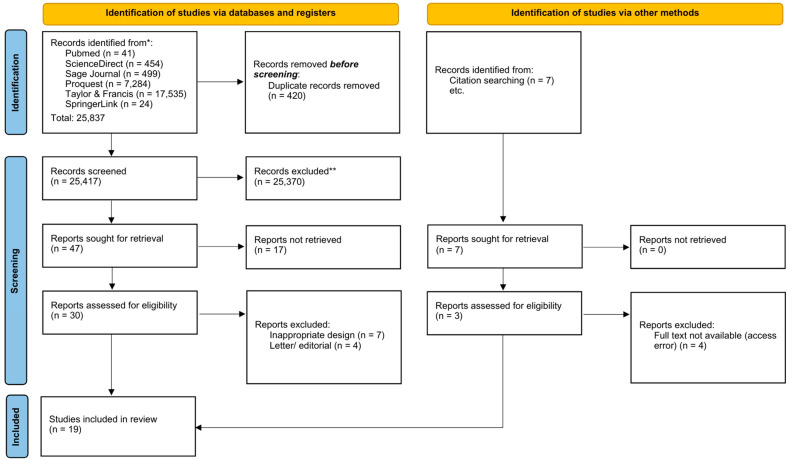
PRISMA (Preferred Reporting Items for Systematic reviews and Meta-Analyses) flowchart. * Indicates the number of records identified from each database or register during the initial search phase. ** Indicates the number of studies excluded during the screening process based on title and abstract assessment, by inclusion criteria.

A literature search across six databases identified 25,837 published articles. Several articles were excluded due to ineligibility as determined by automation tools (*n* = 427). As a result, 25,370 articles were excluded due to non-compliance with the specified study design and inclusion criteria. Subsequently, numerous journals were excluded due to ineligible data, including review articles, books, non-English articles, and articles inaccessible due to subscription-based publication models (*n* = 28). Figure 1 illustrates the PRISMA flowchart. Thus, fifteen articles were included in this systematic review and meta-analysis.

### 2.2. Risk of Bias Analysis

**Figure 2:**
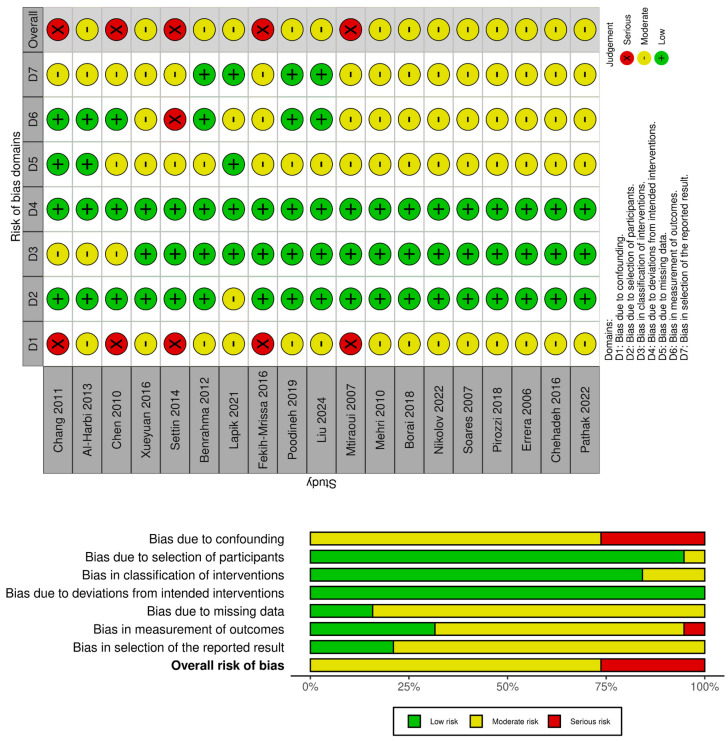
Risk of Bias Analysis using ROBINS-I analysis. Traffic light plot and summary plot.

Figure 2 illustrates a comprehensive risk of bias evaluation across the nineteen studies included in this meta-analysis using the ROBINS-I (Risk Of Bias In Non-randomised Studies–of Interventions) tool. This visualization includes two components:

(a) Traffic Light Plot. This segment provides a detailed visual summary of the bias assessment for each individual study across seven domains: bias due to confounding, bias in the selection of participants, bias in the classification of interventions, bias due to deviations from intended interventions, bias due to missing data, bias in the measurement of outcomes, and bias in the selection of the reported results. Each domain is rated using color-coded indicators as follows: green (low risk of bias), yellow (moderate risk of bias), and red (serious risk of bias). Most studies exhibited a moderate risk (yellow) of bias across the various domains, although five studies showed serious risk (red), predominantly due to unclear reporting of confounding factors and selection processes.

(b) Summary Plot. This summary visualizes the proportion of studies across the entire analysis for each domain of bias assessed. It clearly shows that the majority of the studies demonstrated a moderate risk of bias in multiple domains, particularly concerning bias due to confounding and measurement of outcomes. A small number of studies indicated a serious risk, primarily related to unclear documentation and insufficient explanation regarding participant selection and confounding factors. Overall, although there is some variability in the level of bias, the studies were considered sufficiently reliable to be included in the meta-analytic evaluation, with the acknowledgment of certain limitations in the interpretation of aggregated results due to these biases.

### 2.3. Summary of Included Studies

Table 1 provides a detailed summary of the characteristics of the 19 studies included in this meta-epidemiological analysis, encompassing a total of 6479 participants from four major regions: Asia, Europe, America, and Africa. The specifics of each study include the author name, publication year, country of research origin, number of participants with T2D, control groups (i.e., non-T2D participants), and the distribution of the three rs1801133 polymorphism genotypes (CC, CT, and TT) within each group.

Specifically, Asia is represented by nine studies from countries such as China, Bahrain, India, Iran, and the United Arab Emirates, with T2D patient sample sizes ranging from 56 to 445 individuals and control group sizes ranging from 55 to 350 individuals. Europe includes two studies from Russia and Bulgaria with relatively small sample sizes, consisting of 40–45 T2D patients and 38–40 non-T2D individuals, respectively.

America contributes three studies from Brazil, with T2D patient sample sizes including 25–95 individuals and control groups comprising 16–107 individuals. Finally, Africa is represented by five studies from Egypt and Tunisia, featuring T2D patient sample sizes of 51–67 individuals and control groups including 30–400 individuals.

The distributions of the CC, CT, and TT genotypes are clearly presented, highlighting variations in genotype frequencies across different populations and regions. This information is crucial for subsequent analysis aimed at determining the contribution of each genotype to the risk of T2D and comparing regional and global population risk differences. These data form an essential foundation for understanding epidemiological patterns and the interplay between genetic and environmental factors in the pathogenesis of T2D.

### 2.4. Analysis of rs1801133 Polymorphism

**Figure 3:**
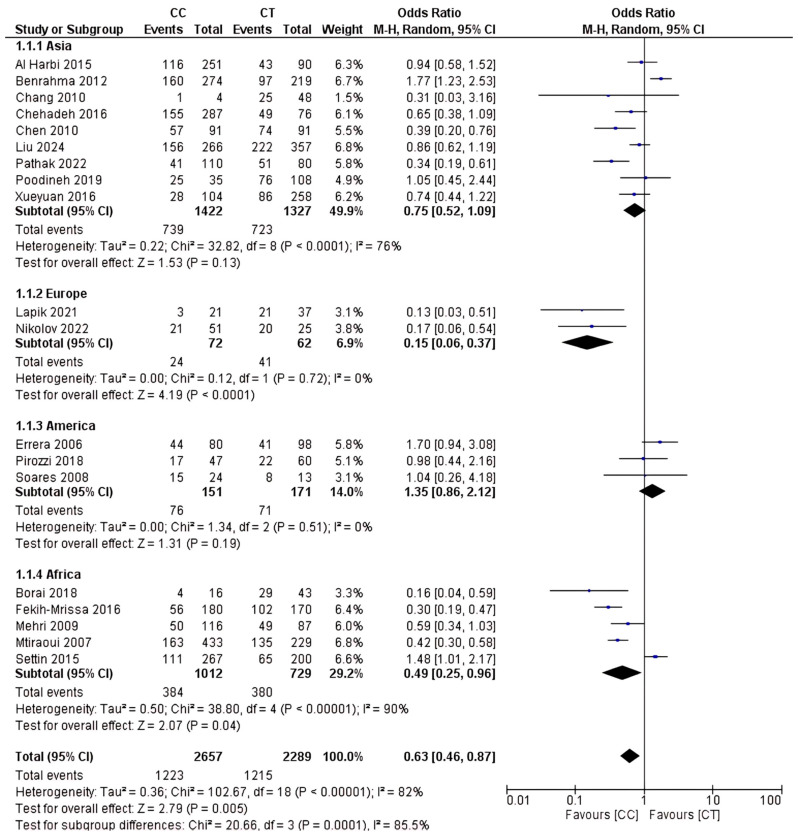
rs1801133 polymorphism; CC vs. CT genotype.

**Figure 4:**
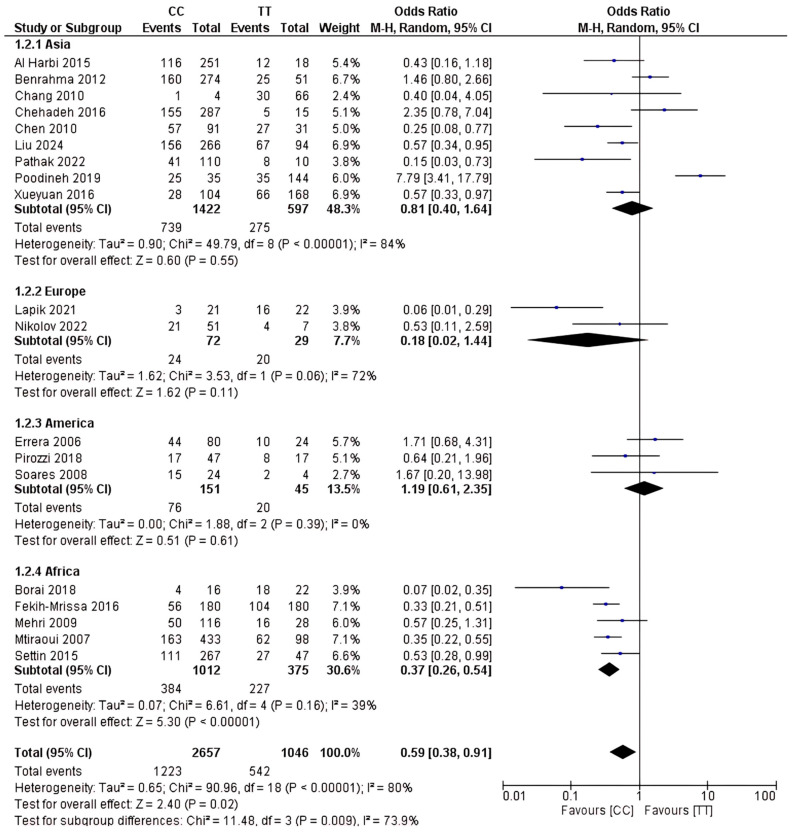
rs1801133 polymorphism; CC vs. TT genotype.

**Figure 5:**
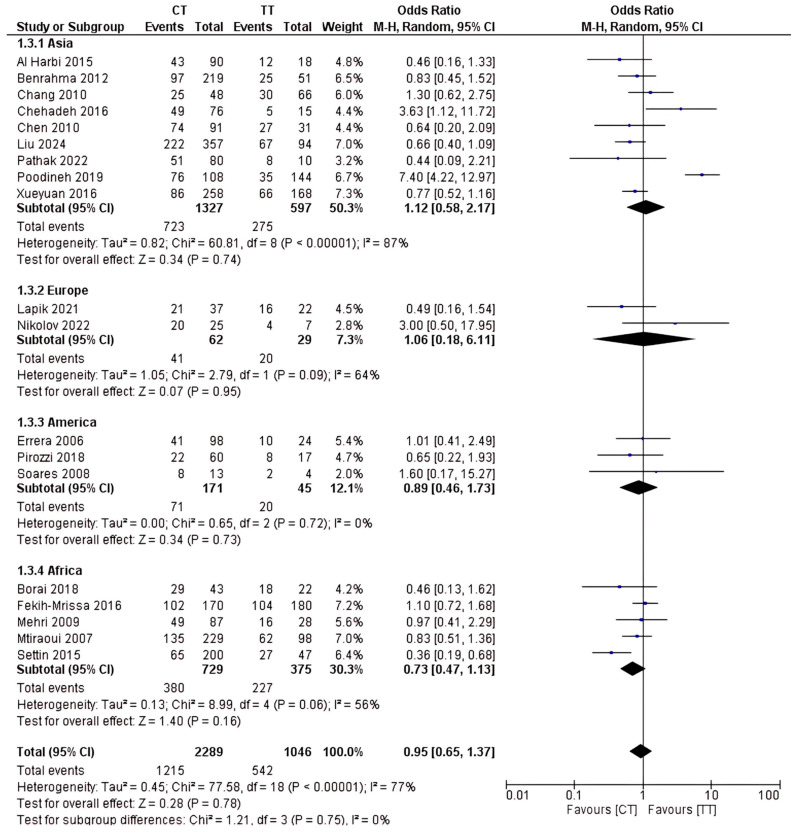
rs1801133 polymorphism; CT vs. TT genotype.

The total sample size included in this study was 6,479 participants. This study investigated how the rs1801133 polymorphism gene is related to T2D risk on a global scale. The results are shown in Figure 3, Figure 4 and Figure 5. Based on this analysis, the CC genotype was shown to be highly contributory to T2D risk relative to the CT and TT genotypes. The results of the CC vs. CT dominant genetic model were OR 95% CI = 0.63 (0.46–0.87); *p* = 0.005; the CC vs. TT genetic recessive model yielded OR 95% CI = 0.59 (0.38–0.91); *p* = 0.02; and the CT vs. TT codominance genetic model gave OR 95% CI = 0.95 (0.65–1.37); *p* = 0.78.

Subgroup analysis revealed that the CC genotype is associated with an increased risk of T2D across several ethnicities. The results of the CC vs. CT comparison for Africa showed OR 95% CI = 0.49 (0.25–0.96); *p* = 0.04, and for Europe, OR 95% CI = 0.15 (0.06–0.37); *p* < 0.0001. In the CC vs. TT comparison for Africa, OR 95% CI = 0.49 (0.25–0.96); *p* < 0.00001. No statistically significant differences were observed in subgroup analysis for either the CT or TT genotype.

## 3. Discussion

**Figure 6:**
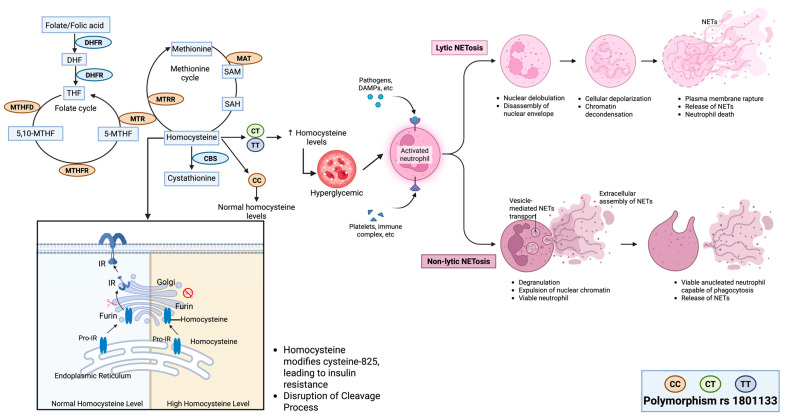
Mechanistic role of MTHFR rs1801133 polymorphism in type 2 diabetes (T2D). The C677T variant alters MTHFR activity, disrupting the folate and methionine cycles, leading to homocysteine accumulation. Elevated homocysteine modifies insulin receptor processing and induces NETosis, promoting insulin resistance and vascular inflammation.

The MTHFR gene plays a crucial role in folic acid metabolism, facilitating the conversion of homocysteine to methionine, which is further processed into S-adenosylmethionine (SAM), a key methyl donor in DNA methylation (Figure 6) [26]. It was found that the rs1801133 polymorphism disrupts homocysteine metabolism, leading to elevated plasma homocysteine levels, which are associated with an increased risk of T2D (Figure 6) [27]. Individuals carrying the T allele of rs1801133 also experience impaired folate metabolism, resulting in higher homocysteine and lower folate levels, which may contribute to metabolic dysfunction and diabetes susceptibility [28,29].

It was found that the rs1801133 polymorphism, with the CC, CT, and TT genotypes, is strongly associated with T2D. Specifically, the TT and CT genotypes are considered risk factors for the disease, whereas the CC genotype is consistently linked to a normal, healthy phenotype [4]. This occurs because individuals with the CT or TT genotypes have higher levels of fasting plasma glucose, homocysteine, and tumor necrosis factor alpha (TNF-α) relative to those with the CC genotype [5]. Surprisingly, our meta-analysis shows that the CC genotype is a risk factor for T2D, whereas other research studies and meta-analyses have indicated that either the CT or TT genotype is associated with an increased risk of T2D [4,30].

TNF-α and fasting plasma glucose are widely recognized in the pathophysiology of T2D, whereas the role of homocysteine is less well known. Homocysteine impacts the insulin system by preventing the cleavage of the proinsulin receptor (pro-IR), resulting in insulin resistance. This is achieved by modifying the cysteine-825 of pro-IR in the endoplasmic reticulum (ER), disrupting disulfide bond formation. The homocysteine-modified pro-IR (C-Hcy) then interferes with interactions with the Furin protease in the Golgi apparatus, impairing the cleavage process required to activate pro-IR [31].

In addition to its effects on insulin signaling, elevated homocysteine levels (hyperhomocysteinemia) contribute to the formation of neutrophil extracellular traps (NETs). Under hyperglycemic conditions, homocysteine further elevates calcium levels and mitochondrial superoxides, accelerating the process of NETosis. This exacerbates vascular complications by promoting inflammation and causing damage to blood vessel walls [32].

### 3.1. SNP Correlation with Type 2 Diabetes

A Single-Nucleotide Polymorphism (SNP) is a genetic variation at a single nucleotide position in DNA, arising from mutations that create base-pair differences. These variations are orthologous, inherited from a common ancestor across generations [33]. Such variations in SNPs can be found in different regions of genes, including promoters, exons, introns, and untranslated regions (UTRs), each of which influences gene expression and function in distinct ways. SNPs in promoter regions can modify transcription factor binding, DNA methylation, and histone modifications, thereby regulating gene activity. Exonal SNPs are categorized as synonymous, such that they do not change amino acid sequences but can influence mRNA stability and translation, or non-synonymous, in which case they directly alter protein structure and function. Intronal SNPs play a role in mRNA splicing, genomic imprinting, and the regulation of long non-coding RNAs (lncRNAs), impacting gene expression at the transcriptional level. SNPs in UTRs influence mRNA stability, translation efficiency, and microRNA (miRNA) binding, further modulating gene expression. These genetic variations contribute to individual differences in traits, disease susceptibility, drug responses, and overall genetic diversity [34].

SNPs are widely studied due to their significant role in influencing genetic susceptibility to complex diseases, including T2D. Since T2D is a polygenic disorder influenced by multiple genetic and environmental factors, SNPs play a crucial role in key biological pathways related to insulin secretion, insulin resistance, and glucose metabolism [35]. Several SNPs have been associated with T2D susceptibility, particularly within the ADIPOQ gene, which regulates adiponectin levels and insulin sensitivity. Notable SNPs, such as rs2241766 and rs1501299, influence adiponectin expression, whereas rs266729 and rs17300539 have been extensively studied for their correlation with insulin resistance and an increased risk of T2D [36].

### 3.2. Dominant Genotype

The results of our analysis indicate that the risk of developing T2D is most prevalent in the Americas, followed by Asia. The rising prevalence of T2D across the Americas is driven by a combination of obesity, poor dietary habits, sedentary lifestyles, socioeconomic disparities, genetic predisposition, and environmental factors. The high obesity rates in Latin America (over 60% of adults classified as overweight or obese) and North America (40% of adults classified as overweight or obese) significantly contribute to insulin resistance. The shift from traditional, nutrient-rich diets to processed, high-sugar foods, coupled with a decline in physical activity due to increasing urbanization and the known increase in desk jobs, has worsened metabolic health across the region [37].

### 3.3. Other Contributing Factors

Epigenetics is defined as a molecular process that modifies reversible gene expression without associated changes in the DNA coding sequences, such as DNA methylation, microRNA, and histone modification [38]. Several external factors (e.g., nutrition, stress, and toxins) are also thought to play vital roles in regulating gene expression. These factors serve as both risk factors and interventional options for gene expression.

It has been shown that bioactive dietary components influence the pathway of DNA methylation by altering the substrates and cofactors necessary for this process, often through modification of the enzyme activity controlling the one-carbon cycle or by transforming DNA demethylation activity [39]. Abnormal methylation variants for controlling food intake are associated with high fat and sugar intakes; this process is thought to be involved in the development of obesity [40]. A mouse model study focusing on a choline-and-folate-deficient (CFD) diet has demonstrated an alteration in hepatic miRNAome profiles. The mice showed pathophysiological and histopathological changes resembling features of human nonalcoholic fatty liver disease. This diet induced the miRNAS expression of miR-134, miR-409-3p, miR-410, and miR-495, together with the activation of hepatic progenitor cells and fibrogenesis in mice with NAFLD-like injury [41]. On the other hand, epigenetic nutrition emerges as a novel alternative to prevent chronic non-communicable diseases. For example, phenol-rich diets are associated with preventing obesity. Another mouse model study assessed the methylation profile of genes involved in adipose tissue triacylglycerol metabolism induced by obesogenic diets versus pterostilbene (a phenol compound known as an antioxidant, commonly found in berries). Obesogenic diets, such as a high-fat and high-sucrose diet, demonstrate an up-regulation of fatty acid synthase (fasn), adipose tissue triglyceride lipase (pnpla2), and peroxisome proliferator-activated receptor γ (pparg). In contrast, pterostilbene reverts the changes induced by an obesogenic diet [42]. 

Additionally, emotional stress has been shown to correlate with the alteration of certain epigenetic processes. A trial using zebrafish models has demonstrated how an unpredictable chronic stressor (UCS) leads to the expression of the pro-inflammatory cytokine genes IL-1β and TNF-α, the anti-inflammatory cytokine IL-10 (negative feedback from the immune system), a reduction in cFOS gene expression, and neuro-inflammation [43]. Furthermore, a study using a xenograft model with transplanted gastric cancer tissue has revealed that chronic stress stimulates the β-adrenergic receptor (ADRB), which leads to the overexpression of VEGF, MMP-2, MMP-7, and MMP-9 in transplanted tumor tissue. Subsequently, this overexpression correlated with tumor size, histological grade, and lymph node metastasis in gastric cancer [44].

Epigenomic alterations are also linked to environmental factors, such as ultraviolet (UV) radiation, γ rays, and genotoxic chemicals. UV radiation is able to modify DNA and RNA methylation patterns. A systematic review on studies evaluates the effect of UV irradiation on HaCat cells (normal human keratinocyte cell line derived from human skin) for the site-specific methylation of p16 and RASSF1 (tumor suppressor genes), demonstrating a hypermethylated profile and decreased transcript levels of tumor suppressor genes, which further contributes to the progression of cellular and tissue degeneration [45].

From both a clinical and public health perspective, the findings of this meta-epidemiological analysis have direct implications for risk assessment and intervention strategies in T2D management. Because rs1801133 polymorphisms interact with environmental and lifestyle factors—such as diet quality, folate intake, physical activity, and obesity—it becomes crucial to integrate them into a lifestyle medicine perspective [6,7,8]. Epidemiological data suggest that regions with a higher prevalence of T2D often exhibit concurrent trends of sedentary behavior and low dietary folate intake [46,47,48], which could exacerbate the functional consequences of MTHFR genetic variants. Consequently, genotype-based risk stratification may inform personalized lifestyle interventions, particularly those targeting homocysteine metabolism through folate-rich nutrition and physical activity. Moreover, the promotion of nutrigenomics-informed prevention, especially in populations with a high prevalence of the CC genotype, could help to mitigate the genetic predisposition toward T2D in a cost-effective and sustainable manner.

The strengths of this study include its large, multiethnic sample size and comprehensive subgroup analysis, providing robust and generalizable findings. Additionally, the detailed exploration of molecular pathways enhances the biological plausibility and clinical relevance of the results obtained, supporting the advancement of precision medicine strategies in diabetes prevention.

### 3.4. Limitation

This study has several limitations that should be acknowledged in order to better interpret its findings. First, although this meta-analysis included diverse populations, many of the original studies lacked allele-level stratification, making it difficult to determine which specific allele (C or T) plays the predominant role in conferring genetic susceptibility to T2D. Second, a substantial proportion of the studies considered employed cross-sectional or case–control designs, which are inherently prone to selection bias and may not adequately account for temporal relationships or residual confounding. Third, differences in genotyping methods, the diagnostic criteria used for T2D, and sample sizes across studies could contribute to heterogeneity and limit the comparability of results. Fourth, environmental and lifestyle confounders—such as diet, folate intake, and physical activity—were rarely adjusted for in the primary studies, although these are known to modify MTHFR function and T2D risk. Finally, although subgroup analyses were conducted, the relatively small number of studies per continent reduced the statistical power of the analyses, especially for Europe and America. Despite these limitations, the overall risk of bias was assessed as moderate, and the consistency of findings across multiple populations strengthens the reliability and generalizability of the meta-epidemiological analysis performed. 

## 4. Method

This systematic review and meta-analysis was meticulously conducted according to the Preferred Reporting Items for Systematic Reviews and Meta-Analyses (PRISMA) guidelines, ensuring rigorous methodological quality and transparency throughout the review process [49]. Additionally, the review protocol underwent external peer review and was officially registered in the International Prospective Register of Systematic Reviews (PROSPERO), which is managed by the National Institute for Health Research (NIHR) under registration number CRD420251009189, further emphasizing our adherence to international standards for systematic review protocols.

### 4.1. Aims and Research Questions

The primary aim of this study was to investigate the associations between the rs1801133 genetic polymorphism and the risk of T2D across global multiethnic populations using a meta-epidemiological approach. The secondary aims were to examine regional differences in genotype distribution and diabetes risk, to evaluate which genotype (CC, CT, or TT) makes the most significant contribution to T2D susceptibility, and to assess the implications of these findings for personalized prevention strategies in different population groups. Based on these objectives, this study was guided by the following research questions:

1. What is the overall association between the rs1801133 polymorphism and the risk of T2D in the global population?
2. Does the contribution to T2D risk differ between the CC, CT, and TT genotypes?
3. How does the strength of association vary across different continents (Asia, Africa, Europe, and America)?
4. What are the potential implications of these genetic findings for population-specific screening and lifestyle-based prevention strategies?

### 4.2. Eligibility Criteria

The inclusion and exclusion criteria were defined prior to the literature search in order to improve the specificity of this review. The inclusion criteria were met by randomized controlled trials and non-randomized studies. The PICO framework underpins the inclusion criteria, which are as follows: (1) population: adults ≥ 18 years old, at risk of T2D (including any specific factors corresponding to the risk, such as high glucose intake, obesity, and metabolic syndrome); (2) intervention: all rs1801133 genotype variants (dominant, recessive, co-dominant); (3) comparison: patients with no risk of T2D and healthy patients; and (4) outcome: T2D. The following were among the exclusion criteria: (1) patients with comorbidities such as gestational diabetes; (2) investigations and trials conducted on non-human subjects; (3) clinical trials using a crossover design; (4) non-English publications; and (5) gray literature.

### 4.3. Search Strategy and Screening

The literature search was performed independently by five researchers (N.A., Y.Y.P.T., A.F.A., A.N., and M.R.A.) until 2 March 2025 using the PubMed, ScienceDirect, SpringerLink, Taylor & Francis, ProQuest, and Sage Journal databases. The primary keywords used in this study were (“Diabetes Mellitus” OR “DM” OR “DM Type 2” OR “Type 2 Diabetes”) AND (“rs1801133” OR “C677T” OR “MTHFR” OR “Methylenetetrahydrofolate Reductase”); these were modified based on the features of each database. 

The article screening process was performed systematically in two distinct stages to ensure accuracy and comprehensiveness in the selection of relevant studies. The initial stage involved a preliminary review of abstracts and titles to efficiently exclude studies that did not align with the predefined inclusion criteria. This was followed by an exhaustive and detailed assessment of the full texts of the remaining articles to rigorously confirm their eligibility. Two independent reviewers (N.A. and Y.Y.P.T.) conducted this evaluation to minimize bias. In instances where discrepancies or ambiguities arose, a third set of reviewers (F.N., R.R.T., and F.M.H.) served as arbiters, collaboratively resolving any disagreements through consensus discussions, thereby guaranteeing a transparent, unbiased, and rigorous research selection process.

### 4.4. Data Extraction and Analysis

Five authors (Y.Y.P.T., N.A., F.N., A.F.A. and A.N.) independently extracted data from the chosen studies into a pre-formatted Google Sheet. This page was prepared to capture crucial study characteristics, including authors, publication year, study design, sample size, patient demographics, risk factor for diabetes, and the type of genotype leading to T2D. The extracted data were then cross-checked by the authors to guarantee accuracy and consistency. Discrepancies in data extraction were resolved by consensus and discussion. The authors of original studies were consulted for clarification or supplemental information as required. 

### 4.5. Risk of Bias Assessment

A risk of bias assessment was conducted on the selected studies utilizing the “Risk Of Bias In Non-randomised Studies–of Interventions” ROBINS-I [50]. The other authors supervised this process. This instrument encompasses seven domains, namely bias due to confounding, bias arising from the measurement of the exposure, bias in the selection of participants for the study (or for the analysis), bias in the classification of the exposure, bias due to missing data, bias in the measurement of outcomes, and bias in the selection of the reported results. The domains were categorized into three groups according to the quality of the study: low, moderate, and serious risk of bias.

### 4.6. Quantitative Analysis

Using Review Manager 5.4, this meta-analysis assessed data using the odds ratio, which includes dichotomous data with a 95% CI (0.05). According to the analysis of these results, random-effects models were used to account for significant heterogeneity and variances in the length of the research. The inverse variance model was used as a statistical technique. Furthermore, I^2^ was used to measure the proportion of total variance attributable to heterogeneity as opposed to chance, and this was applied to quantify heterogeneity. High heterogeneity in the included studies is indicated by an I^2^ value larger than 50%. To evaluate each group’s effect measure and determine the most successful intervention, subgroup analysis was performed on several types of interventions (CC, CT, or TT genotype). To assess the significance of the subgroup analysis results, a significance test was performed among the subgroups; a *p*-value of less than 0.05 was considered significant.

### 4.7. Intervention of Interest

In recent years, researchers have increasingly focused on elucidating the relationship between genetic polymorphisms and T2D, with particular attention paid to the rs1801133 variant. This review aims to provide a comprehensive analysis of the rs1801133 polymorphism in individuals diagnosed with T2D, explicitly examining the distribution and potential implications of the CC, CT, and TT genotypes.

## 5. Conclusions

The conclusions of this comprehensive meta-epidemiological study reveal that the rs1801133 genotype (MTHFR C677T) significantly influences the risk of developing type 2 diabetes mellitus (T2D) globally. This analysis of 19 studies, comprising 6479 participants from diverse ethnic groups across Asia, Africa, Europe, and America, demonstrates that, unexpectedly, the CC genotype is associated with a higher risk of developing T2D relative to the CT and TT genotypes. Subgroup analysis confirms that the CC vs. CT genotype is associated with an increased risk of T2D in both Africa and Europe. In the CC vs. TT comparison, an increased risk was observed in Africa, while no increased risk was found for the CT and TT genotypes

This study also elucidates the molecular mechanisms underlying these associations, notably through disruptions in homocysteine metabolism leading to insulin resistance and vascular inflammation. Furthermore, epigenetic factors, environmental influences, and lifestyle behavior add further complexity to the interactions between genetic predisposition and diabetes risk. These findings emphasize the importance of population-specific genetic screening and the necessity of implementing precision medicine strategies to achieve a more effective preventive intervention for T2D. Further research is recommended to explore diabetes risk based on specific alleles of the rs1801133 genotype, taking into consideration environmental and epigenetic factors and their interactions with genetic predispositions.

## Figures and Tables

**Table 1:** Summary of the studies included.

No.	Author	Country	Samples		Genotypes					
			T2D	Non-T2D	CC		CT		TT	
					Diabetes	Non-Diabetes	Diabetes	Non-Diabetes	Diabetes	Non-Diabetes
***Asia***										
1	Al-Harbi 2015 [9]	Bahrain	171	188	116	135	43	47	12	6
2	Benrahma 2012 [10]	Morocco	282	262	160	114	97	122	25	26
3	Chang 2010 [11]	China	56	62	1	3	25	23	30	36
4	Chehadeh 2016 [12]	United Arab Emirates	209	169	155	132	49	27	5	10
5	Chen 2010 [13]	China	158	55	57	34	74	17	27	4
6	Liu 2024 [5]	China	445	272	156	110	222	135	67	27
7	Pathak 2022 [3]	India	100	100	41	69	51	29	8	2
8	Poodineh 2019 [14]	Iran	136	151	25	10	76	32	35	109
9	Xueyuan 2016 [15]	China	180	350	28	76	86	172	66	102
***Europe***										
10	Lapik 2021 [16]	Russia	40	40	3	18	21	16	16	6
11	Nikolov 2022 [17]	Bulgaria	45	38	21	30	20	5	4	3
***America***										
12	Errera 2006 [18]	Brazil	95	107	44	36	41	57	10	14
13	Pirozzi 2018 [19]	Brazil	25	16	15	9	8	5	2	2
14	Soares 2008 [20]	Brazil	47	77	17	30	22	38	8	9
***Africa***										
15	Borai 2018 [21]	Egypt	51	30	4	12	29	14	18	4
16	Fekih-Mrissa 2016 [22]	Tunisia	160	200	56	124	102	68	104	76
17	Mehri 2009 [23]	Tunisia	115	116	50	66	49	38	16	12
18	Mtiraoui 2007 [24]	Tunisia	267	400	163	270	135	94	62	36
19	Settin 2015 [25]	Egypt	203	311	111	156	65	135	27	20

T2D: type-2 diabetes. CC: homozygous for cytosine, i.e., the individual carries two copies of the C allele. CT: heterozygous, i.e., the individual carries one C allele and one T allele. TT: homozygous for thymine, i.e., the individual carries two copies of the T allele.

## Data Availability

The datasets used and/or analyzed during the current study are available in this manuscript or can be requested from the corresponding author on reasonable request.

## List of Abbreviations

T2D	Type 2 Diabetes
MTHFR	Methylenetetrahydrofolate Reductase
SAM	S-adenosylmethionine
SNP	Single-Nucleotide Polymorphism
miRNA	MicroRNA
lncRNA	Long Non-Coding RNA
UTR	Untranslated Region
TNF-α	Tumor Necrosis Factor Alpha
NETs	Neutrophil Extracellular Traps
ER	Endoplasmic Reticulum
IR	Insulin Receptor
C-Hcy	Cysteine-homocysteinylation
UCS	Unpredictable Chronic Stressor
IL	Interleukin
VEGF	Vascular Endothelial Growth Factor
MMP	Matrix Metalloproteinase
ADRB	Beta-Adrenergic Receptor
PRISMA	Preferred Reporting Items for Systematic Reviews and Meta-Analyses
ROBINS-I	Risk Of Bias In Non-randomised Studies–of Interventions
OR	Odds Ratio
CI	Confidence Interval
PROSPERO	International Prospective Register of Systematic Reviews
DNA	Deoxyribonucleic Acid
